# Association between Endothelial Cell-Specific Molecule 1 and Galectin-3 in Patients with ST-Segment Elevation Myocardial Infarction: A Pilot Study

**DOI:** 10.1155/2022/1723309

**Published:** 2022-11-02

**Authors:** Peng Wei, Xiaoqing Wang, Qiang Fu, Bin Zong, Xuekui Liu, Miaomiao Zhang, Bangming Cao, Ke Zhu

**Affiliations:** ^1^Department of Cardiology, Shanghai Jiao Tong University Affiliated Sixth People's Hospital, Shanghai 200233, China; ^2^Department of Cardiology, Xuzhou Central Hospital, Xuzhou 221009, China; ^3^Department of Cardiology, Xuzhou Clinical School of Xuzhou Medical University, Xuzhou 221009, China; ^4^Experimental Center, Xuzhou Central Hospital, Xuzhou 221009, China; ^5^Department of Cardiology, Yantai Affiliated Hospital of Binzhou Medical University, Yantai 264100, China

## Abstract

The biomarkers galectin-3 (Gal-3) and endothelial cell-specific molecule 1 (ESM-1) reflect endothelial function and inflammation. As a consequence, they play an important role in both the diagnosis and characterization of ST-segment elevation myocardial infarction (STEMI). However, no prior study has explored the association between ESM-1 and Gal-3 in STEMI patients. This study is aimed at determining the ESM-1 and Gal-3 levels in the serum of STEMI patients and then exploring the correlation between the levels of these two biomarkers and their clinical significance in STEMI patients. The participants were divided into two groups: the ST group comprised 35 hospitalized STEMI patients while the control group comprised 24 people with normal coronary arteries. In all the patients, venous blood was taken from the middle of the antecubital fossa. The serum ESM-1 and Gal-3 concentrations were determined using an enzyme-linked immunosorbent assay. The results revealed that the ESM-1 and Gal-3 levels in the STEMI patients were 1.6 and 2.8 times higher, respectively, when compared with the controls (*P* < 0.001). Moreover, the ESM-1 and Gal-3 levels exhibited a positive linear correlation (*r* = 0.758, *P* < 0.001) in the acute STEMI patients. In conclusion, the ESM-1 and Gal-3 levels were found to be significantly elevated and correlated in the STEMI patients. Thus, combining these two biomarkers of endothelial dysfunction and inflammation might be useful for the diagnosis and assessment of STEMI.

## 1. Introduction

ST-segment elevation myocardial infarction (STEMI) is a type of coronary heart disease (CHD) that can be either moderate or severe. It is characterized by a rapid onset, rapid course, and poor prognosis [1, 2]. As a consequence, STEMI is a significant contributor to both morbidity and mortality worldwide. Percutaneous coronary intervention (PCI) is an effective treatment for improving myocardial injury in patients with STEMI. Yet, clinicians and researchers are still actively searching for strategies that can predict and stage STEMI. Here, the detection of key biomarkers correlated with the pathogenesis of STEMI is highly significant with regard to its prediction and diagnosis [3].

The pathogenesis of STEMI and the associated myocardial damage are mostly related to the rupture of the atherosclerotic plaque and the vascular obstruction caused by secondary thrombosis [4]. Cardiac troponin I (TnI) is a highly sensitive and specific cardiac enzyme that is widely used in the clinical diagnosis of acute myocardial ischemia. The level of cardiac TnI rises above the threshold 4–8 hours after the onset of myocardial infarction, reaching a peak some 14–36 hours after the onset [5]. In addition to the biomarker of myocardial necrosis, indicators of endothelial dysfunction and inflammation (i.e., two myocardial injury-related events) can be used as biomarkers of acute STEMI.

Endothelial cell-specific molecule 1 (ESM-1), as a molecule capable of contributing to the progress of pathological atherosclerosis and endothelial dysfunction [6], has been recognized as a potential indicator of endothelial function [7]. In fact, a high serum level of ESM-1 has been used as a novel biomarker for the diagnosis and staging of acute STEMI [8, 9]. Galectin-3 (Gal-3), as a *β*-galactoside-binding lectin, is a new biomarker thought to reflect both fibrogenesis and inflammation. Moreover, it is known to be associated with myocardial injury in cases of myocardial infarction. After Gal-3 knock-out in mice, the infarct regions have been found to robustly express Gal-3, leading to it being regarded as a molecule directly related to early myocardial inflammation following myocardial infarction [10]. In cases of atherosclerosis, a large number of macrophages and foam cells gathered at the site of the atherosclerosis secrete Gal-3 and, therefore, cause it to be highly expressed [11]. Furthermore, Gal-3 promotes the activation, chemotaxis, and aggregation of additional monocytes and macrophages, thereby aggravating the atherosclerosis and resulting in a vicious cycle [12, 13]. Thus, Gal-3 is regarded as a significant biomarker for indicating the progression of atherosclerotic lesions [14]. Moreover, Gal-3 is known to exhibit a positive correlation with the level of coronary stenosis in patients with STEMI [15]. Such evidence suggests that both ESM-1, which is related to endothelial function, and Gal-3, which is related to atherosclerosis, have the potential to serve as biomarkers for STEMI.

Prior studies have demonstrated the additive value of combining biomarkers, including those for myocardial necrosis, myocardial stress, and vascular inflammation, in terms of the prediction and diagnosis of STEMI [16]. However, no association between the ESM-1 and Gal-3 levels has previously been reported in STEMI patients. In light of this, the present study measured the levels of ESM-1 and Gal-3 in the serum of STEMI patients and then investigated their association. It was hypothesized that combining ESM-1 and Gal-3 may result in a valuable biomarker for severe STEMI.

## 2. Materials and Methods

### 2.1. Subjects

This study was conducted in the inpatient clinic of the Department of Cardiology, Xuzhou Central Hospital, China. Approval to conduct the study was granted by the Ethics Committee of Xuzhou Central Hospital. In addition, all the research procedures complied with the requirements set out by the Ethics Committee of Xuzhou Central Hospital and the 1964 Declaration of Helsinki and its subsequent amendments or similar ethical standards. The study involved 35 patients who were initially admitted to the hospital with acute STEMI between January 2018 and December 2019. In total, the patient population comprised 22 males and 13 females with a mean age of 57.25 ± 10.58 years (range: 30–75 years). The control group comprised 24 subjects with normal coronary arteries as diagnosed using coronary angiography or coronary computed tomography angiography.

The inclusion criteria for the study were elevated TnI levels within 12 hours of onset and at least one of the following: chest pain lasting for more than 30 min; an electrocardiogram (ECG) showing ST-segment elevation of two or more thoracic leads or limb leads of ≥0.2 mV or ≥0.1 mV, respectively, and potentially, a left bundle branch block; an ECG showing an abnormal Q wave; loss of myocardial activity or abnormal ventricular wall movement; and/or intravascular thrombosis according to coronary angiography or autopsy [17].

The exclusion criteria for the study were heart function graded III–IV according to the New York Heart Association (NYHA) standards, as complicated by acute or chronic infection or immune system disease (e.g., diabetes, cancer, rheumatism, liver disease, or kidney disease); a history of PCI for the treatment of CHD; and/or a history of cerebrovascular or peripheral vascular disease.

### 2.2. Baseline Information

The participants' name, gender, age, body mass index (BMI), risk factors for cardiovascular disease (smoking history, hypertension history, total cholesterol, triglyceride, low-density lipoprotein cholesterol, and family history of CHD), and levels of creatinine, aspartate aminotransferase, alanine aminotransferase, TnI, and left ventricular ejection fraction (EF) were recorded in both groups.

### 2.3. Detection of ESM-1 and Gal-3 Using Enzyme-Linked Immunosorbent Assay (ELISA)

In all the participants, 5 mL of venous blood was collected via venipuncture at the center of the elbow immediately after the onset of STEMI and admission to the hospital. Following coagulation for 1 h and centrifugation for 15 min at 3000 r/min at 4°C, the serum was then separated from the blood, transferred to an Eppendorf tube, and stored in a -80°C refrigerator before testing. The ESM-1 and Gal-3 concentrations were determined using the ELISA according to the manufacturer's instructions. More specifically, the ESM-1 ELISA kit was purchased from Shanghai Enzyme-linked Biotechnology Co., Ltd. (Shanghai, China), whereas the Gal-3 ELISA kit was purchased from Bender MedSystems (Vienna, Austria).

### 2.4. Statistical Analysis

All the data analyses in this study were performed using Statistical Package for the Social Sciences (SPSS) version 23.0 software (IBM, Armonk, NY, USA). The chi-square test or Fisher's exact probability method was used to analyze the differences between the groups. The count was expressed as the number of cases/percentage (*n*/%), while the measured data were presented as the mean ± standard deviation (SD). If the data for the two groups fitted a normal distribution, an independent samples *t*-test was used for the difference analyses. By contrast, if the data did not fit a normal distribution, the Mann–Whitney rank sum test was used. The Pearson product-difference and Spearman correlation analyses were used for the correlation analysis, with the former being suitable for those variables that conformed to a bivariate normal distribution. Statistical significance was set as a *P* value of less than 0.05.

## 3. Results

### 3.1. Comparison of Baseline Data

There were no significant differences in terms of the participants' gender, age, BMI, smoking history, hypertension history, family history of CHD, or levels of total cholesterol, triglyceride, and creatinine between the two groups (*P* > 0.05). However, the levels of low-density lipoprotein cholesterol, aspartate aminotransferase, alanine transaminase, and TnI were significantly higher in the patients with acute STEMI than in the control group (*P* < 0.05), while the EF value in the acute STEMI patients was significantly lower than in the controls (*P* < 0.05) (Table 1). Elevated serum TnI level is considered to be indicative of STEMI.

### 3.2. Increased Serum ESM-1 and Gal-3 Levels

**Figure 1:**
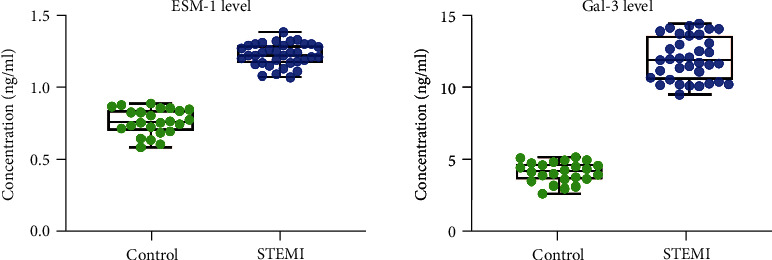
Comparison of the ESM-1 and Gal-3 levels in the two groups measured by the enzyme-linked immunosorbent assay. STEMI vs. Control, both *P* < 0.001.

The patients with acute STEMI had significantly higher serum ESM-1 and Gal-3 levels than the controls (*P* < 0.001) (Table 2 and Figure 1). Moreover, when comparing the concentrations of these two indicators between the two groups, the average ESM-1 concentration was 1.6 times higher (1.23 ng/mL vs. 0.77 ng/mL), and the average Gal-3 concentration was 2.8 times higher (11.86 ng/mL vs. 4.12 ng/mL) in the patients with acute STEMI.

### 3.3. Correlation between Gal-3 and ESM-1 Levels

**Figure 2:**
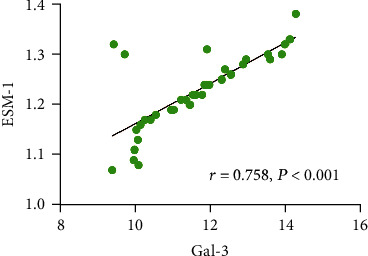
Spearman correlation analysis of the serum ESM-1 and Gal-3 levels in the acute STEMI patients.

A bivariate Spearman correlation analysis was performed to explore the association between the ESM-1 and Gal-3 levels in the acute STEMI patients. The results revealed there to be a positive linear relationship between the ESM-1 and Gal-3 levels (Figure 2, *r* = 0.758, *P* < 0.001), suggesting that when patients have increased levels of Gal-3, they may also have increased levels of ESM-1 in their blood.

## 4. Discussion

The present study compared the serum ESM-1 and Gal-3 levels of venous blood drawn from the center of the elbow between 35 patients with STEMI and 24 controls with normal coronary arteries and then examined the correlation between the concentration changes. It was determined that both the serum ESM-1 and Gal-3 levels were increased in the patients with STEMI. In addition, there was a positive correlation between the ESM-1 and Gal-3 levels.

ESM-1 is recognized as an important marker of vascular endothelial cells [18], while a previous study revealed the correlation between ESM-1 and STEMI [19]. Moreover, in a prior study conducted among STEMI patients with stress hyperglycemia, an ESM − 1 level > 1.01 ng/mL was found to be an independent indicator of adverse cardiac events [6].

There are a few reports in the literature concerning the release kinetics of ESM-1 in STEMI patients, with such studies indicating the ESM-1 levels in patients with acute myocardial infarction to be significantly higher than those in the controls, which is consistent with the present results [20]. Qiu et al. further demonstrated that ESM-1 exhibits positive correlation with the neutrophil-to-lymphocyte ratio as well as with the level of high-sensitivity C-reactive protein (hs-CRP) in STEMI patients with type 2 diabetes mellitus [8]. Furthermore, previous studies concerning the changes in the ESM-1 levels of STEMI patients following the administration of ticagrelor found that the hs-CRP and ESM-1 levels tends to decrease on the fourth and seventh days after administration [21, 22]. These studies confirmed ESM-1 to be an important biomarker and predictor with regard to STEMI.

The inflammatory reaction represents an important process that occurs during pathogenesis and may also be present at the onset of acute myocardial infarction. When experiencing myocardial ischemia, both the local and systemic immune systems are activated and so release different cells and biomarkers able to be detected in the peripheral blood [23]. Gal-3 is one such inflammatory marker. Macrophages are known to be the main cells responsible for secreting Gal-3, although neutrophils, eosinophils, mast cells [8], and fibroblasts also produce Gal-3 [24] in cases of acute myocardial infarction. Previous studies have shown that Gal-3 not only promotes the spread of vascular inflammation by supporting the migration chemotaxis of the monocytes in the vessel wall but also promotes the M1 phenotype of the macrophage subtypes, thereby promoting the instability of the atherosclerotic plaque [25]. In a mouse model of atherosclerosis, Iacobini et al. demonstrated that Gal-3 affects the plaque progression while reducing the effective removal of the modified lipoproteins [26]. In addition, Mac Kinnon et al. found that inhibiting Gal-3 in mice reduces the progression of atherosclerotic plaques [27].

Given the existence of contrasting findings, the secretion kinetics of Gal-3 at the onset of acute myocardial infarction remain uncertain. Animal experimental data have shown that 30 minutes after acute myocardial infarction, the level of Gal-3 messenger RNA begins to increase, whereas a significant increase in the plasma Gal-3 concentration does not occur until 24 hours later [28]. As for the peak level of GAL-3, there appear to be two main time points: one week and 14 days after myocardial infarction [29, 30]. It takes 4–8 hours from the onset of myocardial infarction for the TnI level to increase above the reference threshold, with a peak being reached after 14–36 hours and lasting for 5–7 days [5]. When comparing the peaks of the Gal-3 and TnI levels, it has been speculated that the changes in the myocardial injury kinetics that occur following acute myocardial infarction activate the inflammatory response and so increase the area of myocardial injury. The continuous aggravation of the myocardial injury may lead to the excessive activation of the inflammatory response. Furthermore, some scholars are skeptical regarding the consistency and association of the Gal-3 levels in the peripheral blood and endocardium. Thus, more clinical trials and larger sample sizes are required to elucidate the specific link between them.

The importance of ESM-1 and Gal-3 in terms of the diagnosis and prediction of CHD and STEMI has been proven in several studies. ESM-1 is thought to be a biomarker of endothelial dysfunction, whereas Gal-3 is considered to be a biomarker of atherosclerotic plaque progression and instability [14]. Various biomarkers reflect different pathobiological events following myocardial infarction; therefore, the use of a combination strategy might provide more information for risk assessment than the use of any individual biomarker. However, the relationship between ESM-1 and Gal-3 has not previously been investigated. The statistical analysis performed in this study revealed a linear relationship between the serum concentrations of these two biomarkers (i.e., ESM-1 and Gal-3) in the STEMI patients. That is, the concentration of ESM-1 increased as the concentration of Gal-3 increased. Although the underlying mechanisms of the two molecules remain still unclear, cell experiments have demonstrated that Gal-3 can participate in the phenotypic transformation of vascular smooth muscle cells (VSMCs) [31], a key process associated with the instability and progression of atherosclerotic plaques. This phenotypic transition of the VSMCs from a differentiated to a dedifferentiated state, as well as the involvement of the inflammatory response, may cause the two molecules to interact with each other [32]. Presumably, the sudden rupture of the atherosclerotic plaques leads to the massive release of both ESM-1 and Gal-3, ultimately resulting in the initiation of STEMI.

### 4.1. Study Limitations

While this study has been able to present some prospective findings concerning the correlation between ESM-1 and Gal-3 in STEMI patients, it must be acknowledged that it had several limitations. The first limitation concerns the blood sampling procedure. Intracoronary blood sampling with a high aspiration catheter rather than peripheral blood sampling may be more conducive to ensuring the accuracy of the detected ESM-1 and GAL-3 levels. Second, this pilot study had a small sample size, meaning that it was underpowered. The sample size does not meet the criteria for drawing conclusions regarding a causal relationship, which represents an important limitation of this investigation. Conducting studies with larger sample sizes will improve the persuasiveness of the present results. Moreover, although this study was performed in accordance with safety regulations concerning STEMI patients, there were several related limitations, including certain safety and ethical issues. Therefore, further animal and cell experiments are necessary to further elucidate the role of the ESM-1 and Gal-3 cell signaling pathways in a STEMI model.

## 5. Conclusions

In conclusion, the ESM-1 and Gal-3 levels were found to be increased in the serum of patients with STEMI. Additionally, the elevated serum ESM-1 level was positively correlated with the elevated serum Gal-3 level, indicating there to be an inherent interaction between these two biomarkers during the acute stage of STEMI. The present study is the first to clarify the significant association between the ESM-1 and Gal-3 levels in STEMI patients. Timely and appropriate treatment based on the changes that occur in the levels of these two biomarkers should serve to reduce the mortality rate in patients with STEMI.

## Figures and Tables

**Table 1:** Baseline data for both groups.

Indexes	STEMI (*N* = 35)	Control (*N* = 24)	*t*/*χ*^2^/*Z*	*P*
Gender (male, *n* (%))	22, 62.9%	15, 62.5%	0.008	0.978
Age (years)	57.25 ± 10.58	57.68 ± 10.32	0.155	0.878
BMI (kg/m^2^)	26.24 ± 1.86	25.84 ± 2.31	-0.748	0.458
CV risk factor (*n*[%])				
Smoking history	16 (45.7)	8 (33.3)	0.904	0.342
Hypertension history	11 (31.4)	6 (25.0)	0.287	0.592
Family history of CHD	5 (14.3)	3 (12.5)	0.039	0.844
TC (mmol/L)	4.46 ± 0.90	3.99 ± 1.04	-1.853	0.069
LDL-C (mmol/L)	3.09 ± 0.81	2.54 ± 0.93	-2.501	0.015
TG (mmol/L)	1.69 ± 0.72	1.42 ± 0.52	-1.543	0.128
AST (u/L)	190 (57.0 ~ 284.0)	15.0 (14.0 ~ 18.0)	-6.261	0.000
ALT (u/L)	43 (29.0 ~ 66.0)	19.0 (13.0 ~ 28.0)	-4.715	0.000
Creatinine (umol/L)	54.43 ± 13.83	53.77 ± 15.88	-0.170	0.886
Troponin I (ng/L)	29.16 (11.15~50.00)	0.0 (0.0 ~ 0.03)	-6.427	0.000
EF value	49.37 ± 7.97	58.33 ± 1.58	5.417	0.000

BMI: body mass index; CV: cardiovascular; AST: aspartate aminotransferase; LDL-C: low-density lipoprotein cholesterol; ALT: alanine aminotransferase; TC: total cholesterol; TG: triglyceride; CHD: coronary heart disease; EF: ejection fraction.

**Table 2:** Comparison of the ESM-1 and Gal-3 levels in the two groups.

	STEMI (*N* = 35)	Control (*N* = 24)	*t*	*P*
ESM-1 (ng/mL)	1.23 ± 0.08	0.77 ± 0.09	21.21	<0.001
Gal-3 (ng/mL)	11.86 ± 1.46	4.12 ± 0.70	24.11	<0.001

ESM-1: endothelial cell-specific molecule 1; Gal-3: galectin-3.

## Data Availability

The analyzed data sets generated during the study are available from the corresponding author on reasonable request.
